# Revisiting the role of Annexins in membrane trafficking

**DOI:** 10.1007/s00018-025-05780-z

**Published:** 2025-06-13

**Authors:** Thomas Grewal, Volker Gerke, Jesper Nylandsted, Carles Rentero, Carlos Enrich

**Affiliations:** 1 School of Pharmacy, Faculty of Medicine and Health, University of Sydney, Sydney, NSW Australia; 2 Institute of Medical Biochemistry, Center for Molecular Biology of Inflammation (ZMBE), University of Münster, Röntgenstrasse 16, Münster, Germany; 3 Danish Cancer Institute, Strandboulevarden 49, Copenhagen, Denmark; 4 Department of Molecular Medicine, University of Southern Denmark, J.B. Winsløws Vej 21-25, Odense, Denmark; 5 Departament de Biomedicina, Unitat de Biologia Cel·lular, Facultat de Medicina i Ciències de la Salut, Universitat de Barcelona, Barcelona, Spain; 6 Fundació de Recerca Clínic Barcelona, Institut d’Investigacions Biomèdiques August Pi i Sunyer (FRCB-IDIBAPS), Barcelona, Spain

**Keywords:** Annexins, Membrane trafficking, Ca^2+^, Endosomes, Endocytosis, Exocytosis, Recycling compartment, Membrane repair

## Abstract

Ever since their discovery five decades ago, annexins have been implicated in membrane-related events along endo- and exocytic pathways. Over the years, structural, biochemical and cell imaging studies have revealed that annexins facilitate the organization of membrane domains to allow the formation of tight interactions between membranes destined to fuse. Yet, a comprehensive understanding that would elucidate the molecular characteristics, specific pathways and modes of action in relation to membrane trafficking events for all 12 human annexins is still lacking. By and large, annexins are considered cytosolic proteins that bind to acidic membrane phospholipids upon Ca²⁺ elevation. However, as extended Ca²⁺ stores in the ER, mitochondria and lysosomes continuously exchange Ca²⁺ with the cytosol, significant amounts of annexins remain in contact with membranes for the majority of their intracellular lifespan. Hence, how annexins sense and respond to subcellular Ca^2+^ fluctuations in a dynamic manner and how this influences their localized lipid-binding preferences and functions still needs further clarification. This review examines earlier and recent observations that highlight the involvement of annexins in membrane traffic at the crossroads of endo- and exocytic pathways. The role of annexins in those less known facets such as membrane contact site formation, membrane repair, exosome biology and membrane-less compartments including annexins as RNA-binding proteins will also be discussed. Together, these new avenues strongly imply that annexins serve as important regulators of membrane trafficking.

## Introduction

In order to maintain critical cellular functions, cells have to communicate with their immediate surroundings and respond to changes in their microenvironment. Hence, cells continuously internalize vital nutrients, while simultaneously secreting bioactive molecules. For the intracellular distribution of endocytosed biomolecules, the prevailing dogma considered vesicular trafficking as the primary mechanistic pathway for the transport of lipids, proteins and other molecules between intracellular compartments. This concept has been challenged to some extent with the recent discovery of membrane contact sites (MCS) as novel mediators of lipid, including cholesterol, and ion transfer between organelles. Although this has further complicated the understanding of intracellular trafficking, it also allowed for the incorporation of organelles such as mitochondria and peroxisomes into trafficking circuits, reinforcing the idea that cellular compartments are not isolated and inter-organelle communication is required to ensure a functional cellular entity. In fact, both vesicular trafficking routes and MCS function aside each other and together, serving pivotal roles to distribute molecules and building blocks for many cellular processes and functions. In addition, lipids, such as cholesterol and phosphoinositides (PIs), and ions, in particular calcium (Ca^2+^), can communicate information between organelles, both in physiological and pathological conditions. As outlined below, the Ca^2+^- and lipid-binding properties of annexins not only greatly contribute to vesicular transport in endo- and exocytic pathways, but also other less well-characterized events in membrane biology that have received increased attention in recent years, such as MCS formation, membrane repair mechanism and exosome release. Finally, although not the scope of this review, the RNA-binding properties of annexins and their role in RNA trafficking are briefly discussed.

### Annexins are part of the intricate network of membrane trafficking machineries

The term ‘intracellular trafficking’ in cell biology describes the transport itineraries followed by proteins, lipids or other molecules between different organelles or membrane compartments. In this context, the routes for cell entry include endocytosis at the cell surface and the endocytic pathway to lysosomes. On the other hand, the biosynthetic and exocytic/secretory pathways facilitate the exit of substances from the cell. These two main highways are interconnected with ‘domestic’ roads that link different endo- and exocytic organelles. The necessity to keep this traffic under control is mandatory for a healthy cell and dysregulation of transport routes leading to ‘traffic jams’ is observed in a variety of diseases.

The study of membrane traffic was initiated in the late 60’s and early 70’s by George E. Palade and Christian de Duve, who together with Albert Claude, shared the Nobel Prize in 1974 for unraveling the principles of intracellular membrane traffic [1]. Most important studies early in this period relied on the use of electron microscopy (EM), in combination with the enzymatic analysis of cellular fractions. Together, they resulted in the functional elucidation of the secretory pathway, showing that secretory proteins were synthesized in the endoplasmic reticulum (ER), passed through the Golgi complex, and then were packaged into granules for exocytosis at the plasma membrane. Although endocytosis was the first course of membrane traffic to be recognised earlier, cellular uptake mechanisms only became a central topic in cell biology when endocytosis was proposed to serve as a pathway for the recovery of secretory vesicle components following their insertion into the plasma membrane during exocytosis [2, 3]. From then on, the following two decades were especially fruitful in regards to the identification of the principal routes and the discovery of protein families, such as Rab-GTPases, soluble N-ethylmaleimide-sensitive factor attachment protein receptors (SNAREs) and a subset of Ca^2+^-binding proteins, as the first regulators of intracellular transport pathways [4] that impacted on the cellular homeostasis in health and disease. Research in the field was further reinforced with the identification of vesicle-mediated inter-organelle shuttling of material among most intracellular compartments, except for mitochondria and peroxisomes.

Shortly after unravelling the principles of the secretory pathway, the first annexin was discovered. In 1978, synexin (now known as annexin A7; AnxA7) was found to cause membrane contact/aggregation and fusion of chromaffin granules [5]. In 1982, another Ca^2+^-binding protein, synhibin (AnxA6) was shown to inhibit AnxA7-induced chromaffin granule aggregation and fusion, and accordingly was proposed as an additional regulator of exocytosis [6]. Several other studies then correlated the Ca^2+^-dependent phospholipid-binding behaviour of proteins with particular membrane trafficking steps. It then became clear that these newly discovered proteins were related, with two turning points in the field: the unification of nomenclature [7] and a pioneering article from Carl Creutz that placed the annexin family in the world of intracellular trafficking [8]. It should be noted that in these initial studies, the conclusions drawn on the role of annexins in intracellular trafficking were mostly based on indirect observations, e.g. through their identification in a variety of subcellular fractions, such as chromaffin granules, endosomes, phagosomes or the plasma membrane [9].

Since these earlier findings, it is now well established that annexins are a widely expressed family of proteins containing a variable N-terminal leader followed by a conserved C-terminal domain with a 70 amino acid core sequence repeated four or eight times that mediates the Ca^2+^- and phospholipid-binding properties [10]. Over the years, several of the 12 human annexins have been studied in more detail to dissect and understand the molecular basis, specific pathways, mechanisms and modes of action in the complex landscape of membrane trafficking. Overall, the role of annexins in membrane trafficking along endo- and exocytic routes is best summarized by their ability to mediate the formation of membrane domains supporting docking and fusion events and to facilitate the establishment of tight interactions between membranes destined to fuse [11].

Furthermore, recent advances in our understanding of the biology of annexins have identified several new avenues for further investigation, all related to membrane trafficking. This includes their involvement in the coordination of MCS formation and in the repair of damaged membranes. Accordingly, the identification of these novel functions related to annexins has suggested opportunities for the development of innovative therapeutic strategies [12].

## Annexins in membrane trafficking

As stated above, the capacity of certain annexins to facilitate Ca^2+^-dependent vesicle aggregation in vitro prompted the initial hypothesis for their role in exocytosis [8]. Based on their localization on clathrin-coated pits (CCPs) and endosomes [9, 13, 14], their capacity to mediate early endosome fusion in vitro [9] and the ability of dominant-negative mutants to disrupt endosome distribution in intact cells [15], annexins were considered to also provide critical regulatory functions in endocytosis. In the following, we examine the roles of annexins in regulating trafficking within endo-/exocytic and recycling pathways, with a particular focus on those more elusive facets of annexin biology, which significantly contribute to a better understanding of their multiple functions in membrane trafficking.

### Annexins in endocytosis

The endocytic compartment is a membrane network in functional continuity of the plasma membrane, connecting the extracellular environment with intracellular trafficking stations, facilitating the exchange of information, nutrients, and growth factors [16]. Endocytic vesicles emanating from the plasma membrane fuse with early endosomes, which are the sorting stations for cargo and receptors to be either targeted to late endosomes/multivesicular bodies (LE/MVBs) and lysosomes or returned to the cell surface along the recycling pathway. Although the overall molecular machinery for these routes has been deciphered, the current literature is full of new mechanistic detail related to the fine-tuning of particular endocytic trafficking steps. Moreover, the implication of membrane contacts of endocytic vesicles with the ER for early endosome maturation adds new complexity [17, 18]. In addition, not only coats, Rabs, SNAREs, sorting nexins, PIs and the cytoskeleton, but many other components, including annexins, participate to ensure the correct functioning and sorting/delivery of different cargos. Also, signaling molecules, entailing lipids and proteins, play essential roles in the abovementioned trafficking events.

Early endosomes critically serve as the initial sorting station, receiving membrane material from diverse endocytic vesicles that originate from CCPs, caveolae, macropinosomes and other uptake routes. The early endosomal compartment then orchestrates further membrane and cargo trafficking through the generation of vesicles, tubules, and intraluminal vesicles [19]. As a pivotal step in the endocytic pathway, early endosomes necessitate the presence of membrane-associated regulators to sustain their structural and functional integrity. These regulators are responsible for a range of membrane dynamics activities, including fusion, budding, and tubulation. As outlined below, several annexins contribute to these processes. Finally, during the conversion (maturation) of early endosomes or phagosomes into late endosomes (endolysosomes), the role of certain annexins to regulate the acidification via the vesicular, or vacuolar-type H^+^-translocating adenosine triphosphatase seems to be crucial to facilitate the degradation of cargo.

#### Annexins associated with early endosomes

**Fig. 1:**
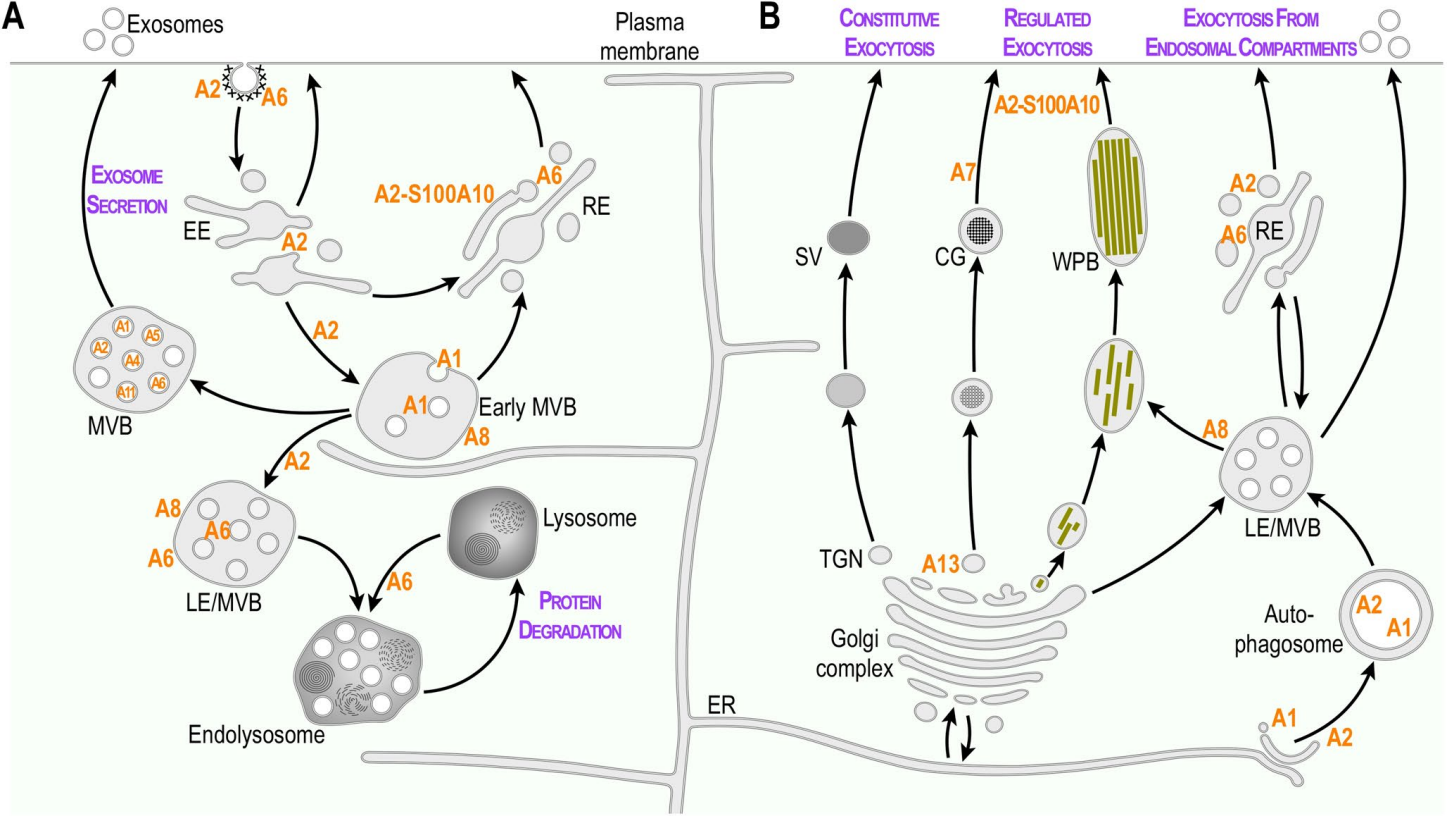
Locations and functions of annexins in endocytic (**A**) and exocytic (**B**) pathways (**A**) The localization and sites of action of AnxA1, AnxA2, the heterotetrameric AnxA2-S100A10 complex, AnxA6 and AnxA8 in endocytic trafficking routes are indicated. AnxA2 and AnxA6 have been implicated in clathrin-mediated internalization steps at the cell surface. AnxA2 regulates early endosomal fusion events, the formation of late endosomes/multivesicular bodies (LE/MVBs) as well as the morphology and distribution of recycling endosomes (RE). AnxA1 contributes to internal vesicle formation within MVBs that contain epidermal growth factor receptor (EGFR). AnxA6 has been implicated in MVB-lysosome fusion. AnxA8 regulates late endosome organization and function. Annexins A2, A5, A1, A4, A11 and A6, ordered according to their abundance in the top 100 proteins in extracellular vesicles (EVs) and exosomes [76] originating from intraluminal vesicles (ILV) of LE/MVBs (see also Fig. 2). (**B**) The contribution of annexins in the regulation of constitutive and regulated exocytosis and exocytic pathways emanating from endosomal compartments is schematically depicted. Biosynthetic flow of cargo, membrane proteins and lipids originates from endoplamic reticulum (ER) membranes and proceeds up to the Golgi compartment simultaneously with glycosylation and processing. The trans-Golgi network (TGN) is the sorting station to different types of secretory pathways. AnxA13 is involved in the formation/transport of apically destined secretory vesicles (SV) at the TGN. AnxA2 in complex with S100A10 and AnxA7 operate in the exocytosis of chromaffin granules (CG) in regulated exocytosis. In endothelial cells, the secretion of von Willebrand factor from Weibel-Palade Bodies (WPB) is also a regulated process, which is triggered upon upon injury or inflammatory activation and requires AnxA2-S100A10. Finally, endolysosomes (LE/MVB) are also able to extracellularly release their contents by means of lysosomal exocytosis, directly or through the recycling endocytic compartment

The first annexin identified on early endosomes was AnxA2, which represents a major component of the machinery facilitating Ca^2+^-dependent homotypic fusion of early and late endosomes [9, 20] and regulating the distribution of early endosomes in cells [15]. Further, Jean Gruenberg’s group showed that AnxA2 is involved in the formation of actin patches on the surface of early endosomes which are crucial for endosome biogenesis. Interestingly, although an F-actin binding protein itself [21], AnxA2 here appears to operate in conjunction with the spire type actin nucleation factor 1 [22]. Importantly, the association of AnxA2 with early endosomes is regulated by tyrosine phosphorylation [23]. AnxA2 was identified early-on as one of the major substrates of src tyrosine kinase with phosphorylation occurring at tyrosine-23 in the unique N-terminal leader domain of AnxA2 [21, 24, 25]. This N-terminal region also harbors the binding site for a unique protein ligand, S100A10, a member of the S100 family of EF-hand Ca^2+^-binding proteins. S100A10 forms a tight dimer and upon binding to AnxA2, triggers the establishment of a heterotetrameric AnxA2-S100A10 complex [26–28]. Thus, cellular AnxA2 protein pools exist in two different physical entities, a monomeric form and a heterotetrameric complex comprising two AnxA2 molecules connected by the S100A10 dimer [21, 29, 30]. Interestingly, only monomeric AnxA2 associates with early endosomes [31], whereas detailed analysis of the subcellular location and characterization of the fate of ectopically expressed AnxA2 mutants revealed that the AnxA2-S100A10 complex is localized to the plasma membrane and the membrane-underlying cortical cytoskeleton [32, 33]. AnxA2 has also been implicated in the regulation of multivesicular endosome or MVB biogenesis [34], and also functions in the secretory pathway, in particular the regulated exocytosis of large secretory vesicles (see below) (Fig. 1A).

Another annexin associated early-on with endosomal trafficking steps is AnxA1 [35]. Colin Hopkins and Clare Futter showed that AnxA1 associated with MVBs and was phosphorylated by the tyrosine kinase activity of the epidermal growth factor receptor (EGFR) upon its internalization into the endosomal system [13]. This association requires Ca^2+^ binding to AnxA1 [35, 36] and the protein was later shown by the Futter lab to be involved in inward vesicle budding of a subpopulation of MVBs that internalize activated EGFR [37]. As shown in Harry Haigler’s and our laboratories, AnxA1 also forms a complex with a member of the S100 family of EF-hand-type Ca^2+^-binding proteins, S100A11, and it can direct this S100 protein to endosomes [38–40]. Interestingly, the AnxA1-S100A11 complex was shown to be involved in the formation of MCS between the ER and endosomes, which function in the transport of cholesterol from the ER to MVBs when the amount of low density lipoprotein (LDL)-cholesterol is low in endosomes [18]. This implicates a role for the AnxA1-S100A11 complex to ensure cholesterol availability required for the inward vesicle budding of MVB subpopulations, but mechanistic details remain to be determined. Endosome-associated annexins also function in the endosomal uptake and escape route of internalized virus. One example is AnxA1 which has been shown to enhance the endosomal trafficking of Influenza A virus (IAV) [41]. Another example is AnxA6, which increases endosomal cholesterol (see below) and thereby impairs IAV replication [42] (Fig. 1A).

Focussing on AnxA6, Stephen Moss and colleagues identified regulatory roles of this annexin in various aspects of cellular signaling and homeostasis, and in particular, the endocytic pathway [43–45]. Several studies in the 90 s then implicated AnxA6 in the first steps of the endocytic pathway, with the Südhof and Anderson laboratories identifying AnxA6 in CCPs, stimulating the budding of CCPs in vitro [46]. Follow-up studies then proposed AnxA6 to recruit the Ca^2+^-dependent protease calpain to cleave the spectrin cytoskeleton, enabling the release of budding vesicles from the plasma membrane [47]. This role for AnxA6 in the remodeling of the actin/spectrin cytoskeleton at the plasma membrane was further emphasized in later studies by Creutz and coworkers, who identified an interaction of AnxA6 with the mu subunit of the adaptor protein 2 [48].

In addition, earlier work in our laboratories recognized AnxA6 in early endosomal fractions from rat liver and together with Rab5, in the apical recycling endocytic compartment [49, 50]. At the plasma membrane and in these early endosomal locations, the spectrin/actin remodeling activities of AnxA6 were proposed to stimulate LDL receptor internalization and probably via a similar mechanisms, the lysosomal targeting of LDL, while transferrin receptor recycling was unaffected [47, 51, 52]. Along these lines, constitutive membrane localization of AnxA6 re-arranged the cortical (F-actin) cytoskeleton [53] and follow-up studies provided the first evidence from live cells for annexins as membrane organizers, showing AnxA6-induced decreased membrane order and plasma membrane remodeling [54]. Also, and similar to other annexin-S100 complexes, the two annexin cores in AnxA6 may allow simultaneous binding to two membranes [55, 56]. In addition, we identified a scaffolding function of AnxA6 for signaling proteins which contributes to determine endocytic receptor trafficking; here AnxA6 acts as a scaffold protein for protein kinase Ca (PKCa) and the GTPase activating protein p120GAP, both negative regulators of the EGFR and its effector, the Ras/mitogen-activated protein kinase pathway, leading to reduced EGFR internalization and targeting to lysosomes for degradation [57, 58]. A scaffolding role for AnxA6 coupled to signaling events in the endosomal recycling compartment probably also exists, as a timely coordination to recycle the Na^+^ -coupled neutral amino acid transporter 4 (SNAT4) to the sinusoidal plasma membrane during metabolic stress triggered by partial hepatectomy was lacking in AnxA6-depleted hepatocytes [59].

#### Annexins associated with the late endocytic compartment

Within the endolysosomal compartment, EM studies have revealed a noteworthy heterogeneity (size, shape, position, electron-density) among late endocytic organelles [60–64]. This marked ultrastructural disparity is a consequence of its position at the crossroads along the maturation process from early to late endosomes, the latter including endocytic carrier vesicles, MVBs, pre-lysosomes, lysosomes and hybrid organelles, but also its close connection with autophagy pathways (autophagosomes, autolysosomes). While cargo degradation has been considered the primary function of this route, current knowledge clearly indicates much more sophisticated and multifunctional roles, including recycling, signaling, cholesterol sorting and lipid metabolism [65, 66]. To achieve these multiple functions, endolysosomes rely on an acidic pH within this compartment, while simultaneously serving as an important intracellular Ca^2+^ reservoir. The relevance of the endolysosomal compartment is further emphasised in circumstances where its functionality is compromised, as observed in lysosomal storage diseases. Indeed, many of these diseases have primary defects in lysosomal functions, especially pH regulation, Ca^2+^ homeostasis and autophagy, affecting autophagosome formation [67].

**Fig. 2:**
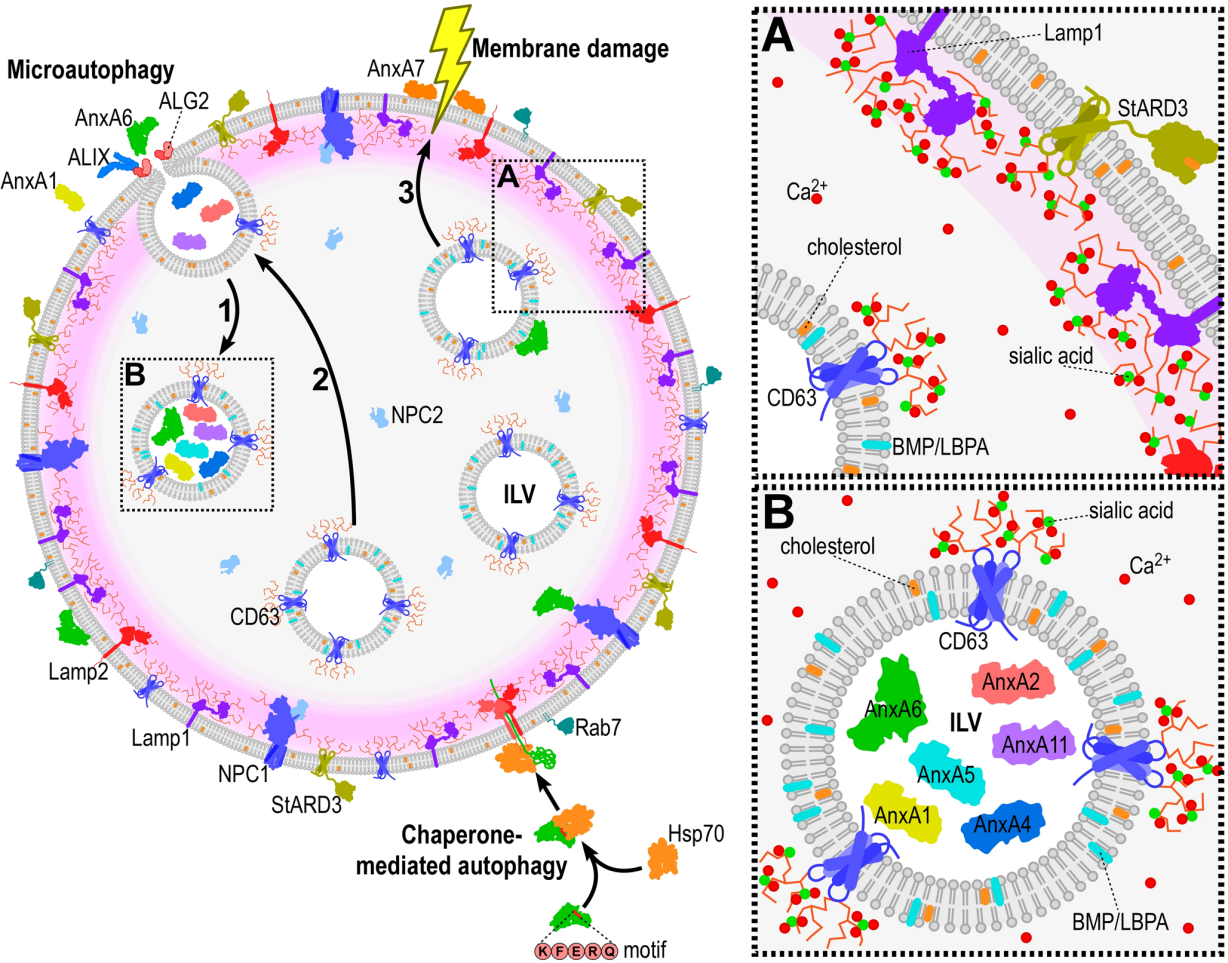
Dynamics of multivesicular bodies and ILV destinations in the endolysosomal compartment. The figure outlines proposed mechanisms contributing to ILV formation and their diversity. The relevance of the endolysosomal glycocalix, generated by resident glycoproteins (i.e. Lamp1/2), as well as the previously underestimated Ca^2+^ reservoir of this compartment (insert A) is highlighted. The four different ILV types are illustrated: the canonical pathway for ILV bearing ubiquitinated cargo (e.g. EGFR) that are destined to lysosomes for degradation (labelled ILV in the scheme); (1) ILVs destined for exosome secretion (illustrated with annexins) (insert B); (2) ILVs implicated in back-fusion with the limiting membrane; (3) ILVs harbouring repair machinery (e.g. AnxA6) and hypothesized to operate in lysosomal membrane repair from the inside. The scheme also illustrates two possible routes by which annexins could get inside the lumen of endolysosomes: (a) via chaperone-mediated autophagy (with Hsp70), or (b) via microautophagy of lysosomal membrane proteins or peripherally bound proteins (for example of the ESCRT-complex: Alix, Tsg101). The location of cholesterol/lipid transporters NPC1/2, StARD3, the phospholipid BMP (LBPA), the tetraspanin CD63, cholesterol and sialic acid is indicated. Abbreviations: Anx, annexin; ALIX, ALG-2 interacting protein X; BMP, bis(monoacylglycero)phosphate (aka, LBPA); EGFR, epidermal growth factor receptor; ESCRT, Endosomal Sorting Complex Required for Transport; ILV, intraluminal vesicles; Lamp 1/2, lysosome-associated membrane protein 1/2; LE/MVBs, late endosomes/multivesicular bodies; Hsp70, heat shock protein 70; NPC1/2, Niemann-Pick type C1/2: StARD3; Star related lipid transfer domain containing 3

In relation to Ca^2+^ homeostasis, several features of LE/MVBs need to be considered. Firstly, LE/MVBs and lysosomes are characterized by the presence of a glycocalyx-like coat that protects the inner lysosomal membrane from degradation. It is composed primarily of rich-branched carbohydrates emanating from the highly sialilated lysosomal-associated membrane proteins 1 and 2, which account for up to 50% of the total endolysosomal proteins. A recent in silico study calculated that the highly negative charge of one sialic acid is capable to bind 2–5 Ca^2+^ atoms. Secondly, intraluminal vesicles (ILVs) contain bis(monoacylglycero)phosphate (BMP; also known as lysobisphosphatidic acid, LBPA), a phospholipid specific to this location and being acidic, also capable to bind Ca²⁺. Thirdly, CD63, a member of the tetraspanin protein family, is enriched in ILVs and exosomes and is also highly glycosylated. Hence, given that the endolysosome represents one of the largest cellular compartments, this signifies an up until recently largely understimated Ca²⁺ store [68]. A variety of Ca^2+^ pumps (Ca^2+^-ATPases), cation exchangers and channels (e.g. transient receptor potential mucolipin 1, two pore channels 1/2) are responsible for maintaining high (∼400–500 μM) Ca^2+^ concentrations inside the endolysosomal lumen [69]. Simultaneously, they create local microdomains on the outside of LE/MVBs facing the cytosol to recruit Ca^2+^-binding proteins such as apoptosis-linked gene 2 (ALG-2), calmodulin and annexins, together with a spectrin/actin coat that includes a variety of actin-binding proteins [70–72] (Fig. 2).

Given the multilammelar structure and complexity of the late endocytic compartment, including the presence of intraluminal vesicles, the exact location of individual annexins within LE/MVBs is not fully understood. Some annexins may be recruited to Ca^2+^-containing microdomains at the limiting LE/MVB membrane, facing the cytosol. Furthermore, annexins may interact with the actin cytoskeleton that is known to coat the LE/MVBs [52, 73] and remain membrane-attached during early to late endosome conversion. Furthermore, annexins were discovered in extracellular vesicles (EVs), including exosomes, with AnxA2, A5, A1, A6, A11 and A4 being among the top 100 proteins identified in exosomes [74, 75]. Hence, an additional scenario needs to be considered regarding the topology of annexins in endolysosomes (see also Fig. 2). Annexins may in fact be inside the lumen of this organelle, at ease with the Ca^2+^ and acidic environment, possibly even associated with the luminal membrane of ILVs. Annexins could enter LE/MVBs by either microautophagy or chaperone-mediated autophagy (CMA) via the ubiquitous pentapeptide targeting motif found in annexins [76]. Once inside the lumen of LE/MVBs, annexins may even assist the back-fusion of ILVs with the endolysosomal limiting membrane. This aspect of annexins, their localization and function within endolysosomes, particularly in LE/MVBs, remains elusive.

The morphological heterogeneity of the endolysosomal compartment is also reflected in the diversity of ILVs and their luminal molecular composition. Either by vesicular transport and fusion from early endosomes, or due to the Endosomal Sorting Complex Required for Transport (ESCRT) -dependent invagination during ILV biogenesis (or microautophagy), different populations of ILVs may co-exist inside. In terms of being found within ILVs, this includes proteins of the ESCRT complex, such as tumour susceptibility gene 101 (Tsg101), one of the most abundant proteins in exosomes, as well as accessory proteins of the ESCRT complex, such as ALG-2 interacting protein X (Alix; for back-fusion) or for endolysosomal membrane proteins, such as synaptotagmin 7 (Syt7), a Ca^2+^ sensor. Once formed, ILVs follow different pathways: (i) ILVs can be targeted to lysosomes for degradation or (ii) undergo back-fusion with the limiting membrane (recycling cholesterol and proteins) [77, 78]. (iii) Furthermore, a population of ILVs can be secreted upon endolysosome fusion with the plasma membrane to become exosomes. Perrin and coworkers [79] suggested that retrofusion (back-fusion) and exocytosis coexist in an equilibrium. Still, several examples illustrate that lysosomally directed and exosomally directed cargoes might be delivered to distinct MVBs [80]. For example, EGF-stimulated EGFRs en route to the lysosome are transported in a subset of MVBs [37], whilst stress-induced EGFRs undergo segregation into a distinct population of MVBs in a manner dependent on the Wiskott-Aldrich Syndrome Protein and SCAR Homolog complex [81].

However, one of the most critical unresolved questions is the identification of MVB populations destined for exosome secretion. To date, the only mechanism operational for the sorting of MVB along secretory or degradative routes is ISGylation, a ubiquitin-like posttranslational modification of Tsg101. This modification has been demonstrated to induce aggregation and degradation, thereby impairing exosome secretion [82].

The mechanism for back-fusion (retrofusion), the fusion of intraluminal vesicles with the limiting LE/MVB membrane, is still poorly understood and only few details of the molecular machinery have yet been uncovered. This route requires an acidic pH and is believed to be mediated by BMP, phosphatidylinositol-3-phosphate and Alix (Fig. 2). It was described that major histocompatibility complex class 2, CD63 and mannose 6-phosphate receptors utilize this particular path to gain access to a variety of locations within the cytosol and then traffic back to the plasma membrane. However, viral and bacterial pathogens such as vesicular stomatitis virus (VSV) or bacillus anthracis also exploit this mechanism to efficiently enter the cytosol of the infected cell. Mechanistically, back-fusion may also require SNAREs or SNARE-like proteins, and yet to be identified tethers, additional proteins and Ca^2+^. Given that the fusion of AnxA2-containing amphisomes, hybrid organelles generated from the fusion of LE/MVBs with autophagosomes, with the plasma membrane has been shown to release AnxA2, a process regulated by Rab8 and Rab27a [83], one may speculate that AnxA2 and possibly other annexins on the luminal side of ILVs may participate in this process.

As outlined in more detail in the previous chapter, AnxA1 is associated with LE/MVBs and together with S100A11, involved in inward vesicle budding in a subpopulation of MVBs [35, 37–39]. Moreover, this heterotetrameric AnxA1-S100A11 complex adds a new function to the annexin family, acting as a tether to enable interorganelle communication via MCS formation between the ER and LE/MVBs (for further details see Chap. 4) [18].

In recent years, our laboratories identified a major role for AnxA6 in late endosomes, relevant for cellular cholesterol homeostasis, with indirect effects on cholesterol-sensitive exocytic pathways, but also directly impacting on the ability of cells to establish membrane contacts between LE/MVBs and the ER [84]. LDL-derived cholesterol loading of LE/MVBs or using genetic or pharmacological inhibition of the late endosomal cholesterol transporter Niemann-Pick type C1 (NPC1), led to the recruitment of significant amounts of AnxA6 to LE/MVBs [51, 85]. Similarly, AnxA6 overexpression also caused late endosomal cholesterol accumulation [86], which triggered cholesterol depletion in other cellular compartments. This imbalanced cellular cholesterol distribution interfered with the trafficking of caveolin and caveolae formation, and lead to dysfunction and mislocalization of several SNAREs, including synaptosomal-associated protein 23 (SNAP23), syntaxin 4 (Stx4) and Stx6, responsible for fibronectin secretion and recycling of integrins, respectively [86–89]. The underlying mechamism for AnxA6-dependent cholesterol accumulation has been unravelled recently, with AnxA6 recruiting the Rab7-GTPase activating protein (GAP) of the Tre-2/Bub2/Cdc16 family TBC1D15 to LE/MVBs, which promotes Rab7 inactivation [84]. Strikingly, in NPC1 mutant cells, AnxA6 depletion increased Rab7 activity, restoring motility of LE/MVBs and rescuing late endosomal cholesterol export. The latter was associated with increased MCS between LE/MVBs and the ER and required the cholesterol transporter Star-related lipid transfer domain containing 3 (StARD3, also known as metastatic lymph node 64 protein; see also Chap. 4). This study for the first time identified an annexin as a regulator of a member of the Rab protein family, considered central hubs for membrane transport and communication between organelles [84].

Finally, AnxA8, a less well-characterized member of the annexin family, has been found associated with the limiting membrane of MVBs, most likely contributing to the coupling of this membrane to the actin cytoskeleton [90]. In endothelial cells, AnxA8 on LE/MVBs was shown to control the delivery of CD63 from LE/MVBs to a specialized lysosome-related organelle, the Weibel-Palade body (WPB), most likely by affecting the distribution of CD63 between ILVs and the LE/MVB limiting membrane. AnxA8 knock-down or knock-out led to a drastic reduction of CD63 levels in WPB and following exocytosis of WPB, to a reduction of CD63 levels on the plasma membrane of activated endothelial cells. As CD63 at the plasma membrane serves to stabilize the leukocyte receptor P-selectin, an important consequence of AnxA8 knock-out was the impaired recruitment of leukocytes to the inflammatory activated endothelial cell surface [91].

Interestingly, loss of AnxA8 also caused a NPC1 mutant-like accumulation of cholesterol in LE/MVBs [92], indicating a positive function, as compared to the inhibitory role of AnxA6 (see above), in cholesterol export from LE/MVBs and lysosomes. This correlated with an association of AnxA8 with cholesterol-rich LE/MVBs and was substantiated in vitro with lipid bilayers, demonstrating that cholesterol reduced the Ca^2+^ requirements of AnxA8 to bind phospholipids such as phosphatidylserine [92]. Similar observations of cholesterol influencing the mode and affinity of phospholipid binding have been reported for AnxA2, AnxA5 and AnxA6 [93, 94], with our laboratories identifying a cholesterol-dependent shift to cooperative phospholipid binding for AnxA2 [95].

#### Annexins in recycling endosomes and retrograde transport pathways

The function of recycling endosomes in various cellular processes, including cell polarity, division, migration and signaling, is well documented. However, the mechanisms by which these endosomes are formed remain to be fully elucidated. Although initially defined as a tubular network that emerges from early/sorting endosomes for the transport of receptors back to the plasma membrane, it is now becoming increasingly evident that the recycling route can also be taken from MVBs, which sort membrane proteins between the recycling and degradative pathways. The segregation of membrane proteins onto ILVs of MVBs results in their removal from the recycling pathway, thereby facilitating their subsequent degradation following the fusion of MVBs with lysosomes. The sorting of numerous cargos onto ILVs is dependent on the ESCRT machinery, although ESCRT-independent mechanisms also exist.

Intriguingly, the recycling endocytic compartment is also passed through by toxins along their retrograde pathway to the Golgi [96]. In fact, studies from the Sandvig and Llorente laboratories provided experimental evidence of a link between the uptake and transport of several toxins and the ability of annexins to bind membrane phospholipids in a dynamic and reversible fashion. Besides the Ca^2+^-binding annexin core domains, this also involved Ca^2+^-related signaling events that trigger dissociation of a complex consisting of AnxA1 and cytoplasmic phospholipase A2α (cPLA2α) [97]. Related to these events, the retrograde trafficking route of shiga toxin is controlled by AnxA1 and AnxA2. While depletion of AnxA1 resulted in increased transport of shiga toxin to the Golgi in a cPLA2α-dependent manner [97], AnxA2 depletion decreased shiga toxin transport [97, 98] (Fig. 1A).

Earlier studies by the Gruenberg and our laboratories had already shown an involvement of AnxA2 in the recycling pathway when analyzing the trafficking and recycling of the transferrin receptor [31, 99]. Recent work by the Delevoye/Raposo group has confirmed and extended the role of AnxA2 in the recycling endocytic compartment, demonstrating that this annexin is part of the molecular machinery involved in the biogenesis of recycling endosomes [100, 101]. More specifically, the formation of tubules extending from early endosomes is aided by the biogenesis of lysosome-related organelles complex 1 (BLOC-1) protein complex, which coordinates the kinesin family member 13 A-dependent elongation of buds into nascent tubules [100]. Once elongated, the nascent tubule probably detaches via local actin polymerization. In these studies, AnxA2-dependent actin polymerization was identified to control the fission of recycling tubules from sorting endosomes, with BLOC-1 acting upstream of AnxA2.

As outlined above (see Chap. 2.1.2), studies from our groups implicated indirected roles for AnxA6 in the recycling compartment, blocking late endosomal cholesterol transport critical for Stx6-mediated recycling of integrins [87]. Most recently, Shin et al. (2024) identified a link between AnxA6 and the delivery of mRNA using lipid nanoparticles, a promising and powerful tool for gene therapy in a variety of diseases [102]. Interestingly, the efficacy of lipid nanoparticle-mediated mRNA delivery can be greatly improved by the use of small molecules that interfere with endocytic recycling, enabling increased release of mRNA from lipid nanoparticles in recycling endosomes. This process could be mimicked by AnxA6 gene depletion, suggesting that loss of AnxA6 functions in the recycling compartment are beneficial for this therapeutic approach [102]. The underlying mechanisms how loss of AnxA6 can provide therapeutic potential when targeting endocytic recycling still need to be clarified. Yet, these studies correlate with earlier findings, identifying AnxA6 as a major protein in purified recycling endosomal fractions from rat liver [14, 50]. Finally, AnxA6 has been shown to facilitate the trafficking of autophagy-related protein 9 A through recycling endosomes, a step that is essential for autophagosomal formation [103].

### Annexins in exocytosis

Since their discovery in the late 70’s, members of the annexin family have been implicated in exocytotic trafficking steps. Following the initial discovery of AnxA7 (synexin) as a protein promoting secretory vesicle contacts in chromaffin cells (see Chap. 1.1) several other annexins were identified in chromaffin granule isolates (then called chromobindins; [104]), suggesting that they shared common properties. A simple model developed at that time for a role of AnxA7 in Ca^2+^-dependent exocytosis proposed that Ca^2+^ triggered the activity of AnxA7 to form contacts between membrane surfaces and the presence of arachidonic acid then promoted the actual fusion event [8]. Although it is now known that other fusion-promoting protein and protein-lipid complexes involving the central SNARE machinery induce membrane fusion in biological systems, a function for annexins in organizing membrane fusion sites and/or bridging membrane surfaces and thereby promoting membrane contact has been shown in many later studies, in particular for AnxA2. Cryo and conventional EM studies revealed that the protein forms junctions between membrane surfaces, in particular in complex with the S100A10 subunit [105], and that it probably constitutes the fine strands observed in EM analysis between chromaffin granules and the plasma membrane of stimulated adrenal chromaffin cells [106]. Biochemical approaches provided further evidence for a role of annexins in exocytosis, in particular Ca^2+^-evoked exocytosis. Using an elegant system of permeabilized chromaffin cells and in vitro granule aggregation and fusion assays, AnxA2 was shown by the Burgoyne and Creutz laboratories to participate in the Ca^2+^-regulated docking and fusion of chromaffin granules [107, 108]. This was later also proven by amperometric and electrophysiological measurements as well as live cell imaging approaches. Importantly, the role of AnxA2 in Ca^2+^-evoked exocytosis was later extended to secretory granules of other cell systems, in particular WPB of endothelial cells and lamellar bodies in alveolar epithelial cells [109–112] (Fig. 1B).

These and subsequent studies revealed that the function of AnxA2 in exocytosis is not only regulated by Ca^2+^, which is required to trigger AnxA2 membrane association, but also by AnxA2-interacting proteins and post-translational modifications. In addition to the src-mediated phosphorylation at tyrosine-23 (see Chap. 2.1.1), phosphorylation/dephosphorylation of AnxA2 at different serine residues in the N-terminal leader sequence has been reported. In chromaffin cells, work carried out in the Bader and Chasserot-Golaz laboratory showed that PKC-mediated serine phosphorylation of AnxA2 was required to promote Ca^2+^-induced secretion of catecholamines by chromaffin granule exocytosis and that this catecholamine release in response to nicotinic stimulation required formation of the heterotetrameric AnxA2-S100A10 complex [113, 114]. Formation of the AnxA2-S100A10 complex was also found essential to support exocytosis of endothelial WPB evoked by adrenergic stimulation and intracellular cAMP elevation. Here, work in our laboratories showed that cAMP/protein kinase A-triggered dephosphosphorylation of AnxA2 by a calcineurin-like phosphatase is a prerequisite for the secretion of von-Willebrand factor through WPB exocytosis. Serine-11 in the S100A10 binding domain of AnxA2 was identified as the target site and dephosphorylation at this residue was essential to permit S100A10 binding, thereby enabling the AnxA2-S100A10 complex to function in promoting WPB exocytosis [115]. As the Anx-type membrane binding sites are accessible in the AnxA2-S100A10 heterocomplex, it can function by bridging two membrane surfaces, thereby supporting tethering. Post-translational mocifications in the N-terminal domain of AnxA2 that inhibit S100A10 binding would interfere with such tethering and a subsequent exocytotic fusion of the granules in question.

However, another membrane bound configuration has also been identified for the AnxA2-S100A10 complex, initially by atomic force microscopy of the complex bound to solid supported bilayers [116]. In this configuration, both AnxA2 subunits were bound to acidic phospholipids in the membrane bilayer and the S100A10 dimer faced the aqueous environment, which would correspond to the cytosol in a cellular context. Here, the S100A10 would be accessible for additional protein interactions, which had been identified in the past by biochemical and proteomic approaches. They include membrane receptors and ion channels but also tethering factors such as Munc13-4, which functions in conjunction with AnxA2-S100A10 in promoting the Ca^2+^-evoked exocytosis of WPBs in endothelial cells [117–120].

Another annexin linked to exocytotic membrane transport is the AnxA13 splice variant AnxA13b, which mediates the transport of sphingolipid- and cholesterol-rich vesicles to apical membranes [121] (see Chap. 3.2). The picture emerging from these studies is that proteins of the annexin family fulfill functions both in the constitutive as well as the regulated exocytotic route, most likely by organizing (plasma) membrane domains for subsequent fusion and/or tethering them to the respective secretory vesicles (Fig. 1B).

## Annexins in membrane trafficking of polarized cells

In general, studies addressing membrane trafficking in mammalian cells need to consider the ability of certain cell types to polarize, which is defined as the asymmetric distribution of lipids and proteins in the plasma membrane. While fibroblasts represent the prototypical non-polarized cell type, the ability of cells to develop an apico-basal polarity provides a critical prerequisite for the development of multicellular tissues, such as epithelia and endothelia. Hence, investigating membrane trafficking in polarized cells entails experimental challenges, for example when trying to compare and consolidate in situ cell analysis within organs and tissues with the limitations provided by the rather two-dimensional analysis of cells in culture. The latter provides some cell polarity, with the basal membrane contacting glass, plastic, or substrate and the apical side facing the culture medium. Further challenging the analysis of polarized cells, the culture of cells from the intestine, liver, kidney or neurons commonly leads to the loss of features related to their apico-basal polarity. In the case of Madin-Darby canine kidney (MDCK) cells or CaCo-2 cells, the use of transwell filters as substrates allows for the maintenance of a well-defined polarity, and for many researchers, served as a model to investigate membrane trafficking in polarized cells.

The cell surface of epithelial cells is differentiated into apical and basolateral plasma membrane domains that are separated by tight junctions [122–125]. The distinctive protein and lipid composition of each domain is generated by the sorting of components into distinct classes of vesicles in the trans-Golgi network (TGN), which then are shipped along different routes to the apical and/or basolateral side [126]. Based on the membrane trafficking studies in these models it was revealed that in particular the apical and transcytotic pathway connecting the apical and basolateral cell surface involve unique factors that provide specificity in the sorting and delivery of membrane components in epithelial cells [127].

Annexins are highly expressed in epithelial cells exhibiting well-differentiated basolateral and apical membranes. This includes hepatocytes as well as cells in the endothelium, muscle, kidney, intestine, pancreas, but also neuronal cells defined by their two distinct extension entities, axons and dendrites. In the following we will summarize the current understanding how annexins contribute to the formation, function, and maintenance of polarized cells in hepatocytes, MDCK (as the prototypic epithelial cell) and neuronal cells. In addition, at least three annexins, AnxA2, A6 and A13b were shown to participate in transcytosis and/or polarized membrane trafficking in hepatocytes and MDCK cells [49, 128, 129] (for further details see below).

### Hepatocytes

Hepatocytes constitute approximately 70–80% of the total liver volume and represent the parenchymal cells, whereas the remaining 20–30% are comprised of non-parenchymal Kupffer, endothelial and stellate cells. The hepatocyte is a highly polarized epithelial cell with a plasma membrane being divided into three main functional domains: the sinusoidal domain, which faces the blood and the hepatic endothelial cells; the lateral domain, which contains junctional complexes such as desmosomes and gap junctions; the canalicular plasma membrane, which is located at the apical pole and plays a role in bile secretion [130].

Based on the biochemical and ultrastructural analysis of highly purified early, late and recycling endosomal fractions from rat liver [131–133], earlier studies identified AnxA6 as a major protein of these endosomes [14, 50, 134, 135], together with several other annexins, including AnxA1, A2 and A4 [50]. Analyzing purified endosomes by immuno-EM, we demonstrated that the majority (80%) of AnxA6 was associated with endocytic structures surrounding the bile canaliculus at the apical domain, while the remainder of AnxA6 was found on subsinusoidal early endosomes. AnxA6 colocalized with substantial amounts of Rab5-positive apical and transferrin-containing endosomes, while no significant colocalisation with the polymeric immunoglobulin receptor, a marker for the transcytotic pathway, was detected [49].

Hepatocytes use the indirect route for the transport of proteins and lipids from the TGN to the cell surface, i.e. proteins are sent first to one surface, usually the basolateral, and from there, the proteins are endocytosed and delivered to early endosomes. Then, proteins can either recycle to the surface of origin, be transported along the endocytic route for degradation in lysosomes or be transcytosed to the apical domain. Hence, these earlier EM studies pointed at roles for AnxA6 within the apical ‘early’ endocytic compartment of the hepatocyte possibly related to receptor recycling and transport to late endocytic/lysosomal compartment pathways [49, 134].

AnxA6 is one of the most abundant proteins in the liver and recently, we investigated the impact of AnxA6 deficiency on liver regeneration, using partial hepatectomy in AnxA6-KO mice as a model. As part of this pleiotropic process, quiescent hepatocytes of the remnant liver enter the G1 phase of the replicative cycle. In order to restore the original hepatic mass, the cellular machinery that governs endo-/exocytic pathways and signaling cascades to maintain feedback control and liver functionality needs to be re-established. Strikingly, AnxA6-KO mice lacked the capacity to internalize alanine into the remnant liver. Consequently, as alanine represents an essential substrate for gluconeogenesis during liver regeneration, AnxA6-KO mice died from hypoglycemia after surgery [59]. Mechanistically, AnxA6-KO hepatocytes were characterized by an inability of SNAT4, the hepatic alanine receptor, to recycle to the blood-facing basolateral plasma membrane after partial hepatectomy, impairing alanine uptake and, consequently, glucose production. The underlying mechanism remains to be identified, but as described earlier (see Chap. 2.1.2), AnxA6 levels indirectly modulate several cholesterol-sensitive SNARE complexes, including SNAP23, Stx4, and Stx6, all with major regulatory roles in exocytic membrane transport routes and involved for example in the transport of SNAT4 [88, 89]. Alternatively, AnxA6 interacts with members of the TBC protein family to control Rab7 activity [84], the latter being involved in the regulation of retromer, a multi-protein assembly that controls endosomal retrieval and recycling [136]. Hence, the lack of AnxA6/TBC protein interaction in the AnxA6-KO mice may alter retromer function and contribute to SNAT4 mislocalization after partial hepatectomy in hepatocytes. Moreover, the multiple scaffolding functions of AnxA6 in several signaling pathways [57, 58, 130, 137, 138] may also contribute to control hormone‐dependent SNAT4 translocation to the plasma membrane. Taken together, AnxA6 may exert multiple functions to control hepatocyte trafficking pathways [49, 134].

### MDCK cells

In comparison to hepatocytes, epithelial cells such as MDCK exploit predominantly the direct route that delivers proteins and lipids from the TGN to the apical membrane. The comparison of basolateral and apical pathways [126] identified Rab8 as highly enriched in the basolateral pathway and involved in transport to the basolateral cell surface [139]. Two isoforms of AnxA13 are expressed in epithelia, with a myristoyl amino-terminal modification and Ca^2+^ differentially contributing to the membrane association of these annexins to sphingolipid- and cholesterol-rich subdomains of the TGN [121], apical and basolateral membranes. The AnxA13b isoform is enriched in the apical pathway [129], in particular at punctate structures underneath the apical surface of MDCK cells. Both AnxA13 isoforms stimulate apical transport of IAV hemagglutinin, yet only AnxA13a inhibits basolateral transport of VSV-G protein, indicating localized and specific roles for the AnxA13 isoforms in epithelial cells [140].

Together with the Mostov group, we utilized MDCK cells to study epithelial morphogenesis, allowing gel-embedded cells to form spherical epithelial monolayers enclosing a central lumen. This study identified apical AnxA2 determining the formation of the apical surface, a fundamental, yet poorly understood step to establish polarity in epithelial organ development [141]. During this process, the segregation and differential distribution of PIs, in particular phosphatidylinositol (4,5)-bisphosphate (PIP_2_) and phosphatidylinositol (3,4,5)-triphosphate (PIP_3_), critically determine apical and basolateral membrane identity. During epithelial morphogenesis, phosphatase and tensin homolog (PTEN) at the apical membrane mediates the generation and enrichment of PIP_2_ from PIP_3_, the latter segregating to the basolateral surface. Strikingly, at the apical membrane, AnxA2 then binds to PIP_2_, which is followed by recruitment of the Rac/Rho GTPase Cdc42 by the AnxA2-S100A10 complex and subsequent association of aPKC with Cdc42. In this series of events, the initial binding of AnxA2 to PIP_2_ at the apical side is critical, as loss of AnxA2 prevented normal development of the apical surface and lumen [141].

Overall, studies from our and other laboratories point at plausible roles for annexin-mediated membrane-membrane interactions in apical transport. This includes AnxA6 in the hepatocyte along the indirect route and AnxA13b in direct route transport of MDCK cells [129, 134]. Yet, more work is still necessary to fully integrate and understand whether annexins are tethers or scaffolding proteins that conribute to the SNARE-dependent targeting and delivery of cargo in polarized cells.

### Neurons and other cells of the central nervous system

Neurons are highly polarized cells, with the axon and nerve terminals representing the apical domains and the cell body and dendrites being equivalent to the basolateral membrane. In neurons, protein trafficking is of critical importance in maintaining cellular homeostasis and the detrimental consequences of its dysregulation are exemplified in the development of several neurological diseases. These include Alzheimer’s, Parkinson’s and Huntington’s disease, Down syndrome, amyotrophic lateral sclerosis and prion disorders, all of which with defects in protein sorting and membrane trafficking [142, 143]. While dysregulated Golgi sorting and the perturbation of Golgi morphology can contribute to neuropathology [144, 145], defects in endolysosomal trafficking and degradation can also lead to the emergence of neurological diseases [146–148]. Although the understanding of neuronal lipid composition and how this may alter brain function is still limited, there is increasing evidence that lipid transport is critical for neuronal homeostasis. This is exemplified by pediatric lysosomal storage disorders such as Gaucher’s or NPC disease, which are characterized by lipid accumulation in LE/MVBs and lysosomes. Similarly, other neurodegenerative diseases such as Parkinson’s and Alzheimer’s, display lipid transport defects associated with lysosomal dysfunction, which is ultimately responsible for the loss of cellular integrity and cell functioning. Along these lines, in the last decade, lipid transfer between organelles through MCS has emerged as a new paradigm. This correlates with compromised lipid transfer across MCS and organelle dysfunction in cell and animal models of NPC [149, 150]. Dysregulated MCS formation, although not necessarily related to lipid transfer, has also been observed in Parkinson’s, Alzheimer’s, Charcot-Marie-Tooth disease, and several other neurological and lysosomal storage disorders [151, 152].

Over the years, annexins have been suggested to exert diverse roles in neuronal development, and central nervous system (CNS) functioning, with links to neurological disorders and CNS tumours. Although the molecular details on the contribution of annexins to normal and dysregulated neuronal functions have yet to be clarified, their ability to participate in membrane organization and to modulate cell signaling is likely relevant in various CNS processes, including endo-/exocytosis, and plasma membrane microdomain formation, with consequences for differentiation, proliferation, and synaptic formation in CNS tissues.

AnxA2 is highly expressed in neuronal growth cones, while in murine neurons, AnxA6 is upregulated during embryonal development, which correlates with AnxA6 upregulation during maturation of hippocampal cultured neurons [153]. AnxA6 then becomes concentrated at the base of the neuronal axon, the axon initial segment (AIS) [153, 154] and almost all neurons at late stage maturation exhibit AnxA6 accumulation in the AIS. Interestingly, the microtubule-associated protein tau interacts with the core domains of AnxA2 and AnxA6 in a Ca^2+^-dependent manner. The localization of these interactions could be relevant during neuronal development, when tau becomes enriched in the axon [155]. In tauopathies that include a group of heterogeneous neurodegenerative conditions such as Alzheimer’s, tau redistributes to the somatodendritic compartment. Indeed, in primary cortical mouse neurons, competing with tau-annexin interaction compromised the axonal enrichment of tau [156]. These findings implicate that dysregulation of tau-annexin interactions [155] could contribute to tau mislocalization and tauopathies.

In neuronal cells, other scenarios connected to AnxA6 and its interaction partner prolin-rich tyrosine kinase 2-beta (Pyk2) may exist [157, 158]. Pyk2 is enriched in hippocampal neurons and transduces a variety of Ca^2+^-related signaling events linked to synaptic plasticity and compromised in neurodegenerative diseases such as Alzheimer’s disease [159], Huntington’s disease [160] or other neurological and psychiatric diseases [161, 162]. Hence, one can speculate that a Ca^2+^-mediated cross-talk between AnxA6 and Pyk2 could play a part in the disrupted Ca^2+^ homeostasis observed in neurodegenerative diseases.

## Annexins and membrane contact sites: formation and functioning

The eukaryotic cell represents a structural and functional unit characterized by organelles that are surrounded by membranes, providing segregation of specific cellular functions. This compartmentalization necessitates the establishment of organelle communication to properly respond to changes in the cell’s microenvironment. In the past, it was generally believed that diffusion or active transport through the cytoplasm, and vesicular trafficking would permit the exchange of signals and metabolites between cellular compartments. However, over the past decade, the identification and significance of MCS for inter-organelle communication and cellular homeostasis is increasingly recognized. Contact sites between different organelles have been identified in all eukaryotic cells examined, acting as platforms for protein interactions, signaling events, lipid exchange and calcium flux. Moreover, recent advances in the field of MCS biology have revealed the importance of non-vesicular communication in orchestrating the coordination of physiological events in order to maintain cellular homeostasis [163, 164]. It is perhaps the transfer of lipids across MCS, in particular cholesterol and PIs, which has been the most striking and new concept in cell biology, leading to an overhaul of existing models on lipid transport between organelles that were predominantly based on cytosolic lipid transporters and/or vesicular trafficking. MCS-mediated communication now also brings organelles such as mitochondria and peroxisomes, which prior to the identification of MCS lacked connectivity to other organelles via vesicular routes, into trafficking circuits to other cellular sites.

In line with their structural and Ca^2+^-inducible membrane-binding properties, annexins appear to fulfill criteria required to bring neighbouring membranes into close proximity. Three annexins have already been identified in MCS to date: AnxA1, A6 and A11. Intriguingly, their participation in the regulation of MCS formation were all discovered in the vicinity of the endolysosomal compartment, with AnxA1 and AnxA6 participating in the contacts between LE/lysosomes and ER and AnxA11 serving as a tether for membraneless RNA granules to lysosomes [163].

The first annexin discovered in MCS was AnxA1. Studies by Emily Eden and Clare Futter demonstrated AnxA1 to act as an anchor/tether connecting the ER and LE/MVBs [18, 165]. During the targeting of EGFR for lysosomal degradation, EGF-induced AnxA1 phosphorylation is crucial for the segregation of EGFR into ILVs [37]. Tyrosine-phosphorylated AnxA1, in conjunction with its binding partner S100A11, functions as a docking site for protein tyrosine phosphatase 1B (PTB1B), an enzyme that is localised to the ER and required for dampening EGFR signaling. PTB1B not only dephosphorylates EGFR, but also enables the ligand-stimulated sorting of EGFR into ILVs at LE/MVB-ER contacts. Hence, LE/MVB-ER contacts may provide localized sites where the phosphorylation state of components of the AnxA1 and MVB sorting machinery could be tightly controlled [13, 18, 37]. Importantly, in settings when cholesterol levels in LE/MVBs are diminished due to reduced availability of endocytosed LDL-cholesterol, these AnxA1-regulated membrane contacts facilitate the transfer of cholesterol from the ER to LE/MVBs [18]. Thereby, the tethering function of AnxA1 ensures the proper functioning of a cholesterol-sensitve and critical step during ILV formation to spatially downregulate EGFR signaling. The cholesterol transfer during this process is facilitated by the interaction of ER-localised VAMP-associated proteins (VAP) and endosomal oxysterol-binding protein 1 L-related protein [18].

The kinetics of AnxA1-S100A11 recruitment and dynamics of protein-protein and protein-lipid interactions to support the formation of MCS in LE/MVB subpopulations for EGFR downregulation have yet to be clarified. However, their MCS-related mode of action may reflect a common theme within annexins. The reversible membrane binding capacity of annexins could establish initial protein-protein (or protein-phospholipid) interactions between LE/MVBs and ER membranes to induce the formation of MCS and allow the exchange of ions and lipids, including cholesterol [84, 163, 166, 167]. The ability of S100 proteins to interact with annexins may then provide additional tethering stability between two membrane compartments [167, 168]. Besides AnxA1, potential candidates include AnxA2, which, in conjunction with S100A10, has the capacity to bind to cholesterol-rich LE [34].

AnxA6 was the second annexin to be identified in MCS, being part of hepatic MAMs [169], specialized membrane subdomains that facilitate communication between the ER and mitochondria. In addition, we recently identified that AnxA6 depletion alleviated late endosomal cholesterol accumulation in NPC1 mutant cells [84]. As outlined above (Chap. 2.1.2), AnxA6 associates with cholesterol-rich endosomes and recruits the Rab7-GAP TBC1D15, which promotes Rab7 inactivation. Consequently, depletion of AnxA6 or TBC1D15 increased Rab7-GTP levels in NPC1 mutant cells, which besides restoring mobility and peripheral distribution of late endosomal vesicles, was also accompanied by increased MCS formation between LE/MVBs and the ER. The latter enabled cholesterol transfer in a StARD3-dependent manner to the ER and subsequent cholesteryl ester storage in lipid droplets. Thus, the levels of AnxA6 in LE/MVBs appear to control communication and contact formation between LE/MVBs and the ER when NPC1 is lacking, identifying novel options to bypass cholesterol transport defects in NPC1 deficiency.

The third annexin to act as a tether related to endolysosomes is AnxA11, which connects lysosomes with RNA granules, functioning as a handle to transport/move RNAs to various cellular destinations [170]. In these studies, the unique AnxA11 N-terminal domain upon undergoing phase transition facilitated Ca^2+^- and phospholipid-dependent lysosome-RNA granule interactions. This mechanism could be relevant for microtubule-based transport of RNA granules in polarized epithelial cells such as neurons, enabling protein translation at different subcellular locations. As contacts between RNA granules and lysosomes and neuronal RNA granule transport are compromised in AnxA11 mutations associated with amyotrophic lateral sclerosis (ALS), this annexin provides a novel structural link between lysosomes and a membraneless compartment in ALS pathogenesis.

Although the identification of annexins in MCS is limited to three members thus far, it is tempting to speculate that due to the high degree of sequence and structural homology within the family, other annexins may also contribute to the formation, maintenance and/or function of MCS. This hypothesis is based on the premise that annexins could possess a common domain or motif that may facilitate binding between membranes. In support of such a model, the analysis of the AnxA6 sequence identified two possible FFAT motifs (two phenylalanines in an acidic environment), which were originally recognized in endolysosomal proteins that interacted with positively charged ER-associated VAPs (VAP-A/B) and the major sperm protein domain of members of the motile sperm domain-containing protein (MOSPD) family. These sequences could be relevant for the localization of AnxA6 in MAMs observed in the liver of mice [169]. Reflecting the complexity of the protein composition in MCS, further analysis of the VAP interactome (VAPome) has identified a large number of proteins containing FFAT sequences [171]. In addition, variations of the FFAT motif have also been identified, including phospho-FFAT and FFAT-neutral domains [172], altogether further increasing the number of potential candidates for MCS. Possibly relevant for annexins is the FFNT motif, a FFAT motif variation present in all human annexins with probability scores to act as tethering motifs comparable to the FFAT motif in the cholesterol transporter and well known tether StARD3 to interact with VAP-A/B or MOSPD2 in the ER. Hence, although experimental evidence for this hypothesis is still lacking, annexins are plausible tether candidates in MCS formation.

Finally, post-translational modifications such as palmitoylation should also be considered. For example, AnxA1 and AnxA6 palmitoylation in EVs and exosomes has been reported [173], and therefore the contribution of palmitoylated annexins in MCS formation cannot be ruled out [174].

## Membrane repair

Maintaining plasma membrane integrity is essential for cell survival and homeostasis. Cells exposed to mechanical stress, such as those in muscle, vasculature, and epidermis, as well as cells challenged by pathogens or chemical damage, are particularly prone to membrane injuries. To counteract these threats, cells rely on highly efficient repair systems that respond swiftly to membrane breaches to restore function [175–177]. As outlined below, members of the annexin protein family are central to these repair mechanisms. Recent studies have highlighted their specialized roles in membrane repair, showcasing their ability to sense damage, bind to phospholipids, and cross-link and reshape membranes to promote healing [178–181].

### Annexins and plasma membrane repair

In mechanically stressed cells, rapid plasma membrane repair is critical to prevent cytoplasmic loss and preserve cell function. Annexins are key players in this process, capable of binding to damaged membranes and facilitating their resealing. By inducing membrane curvature and cross-linking phospholipids, annexins repair and stabilize damaged regions, which is essential for maintaining membrane integrity under stress [178–183].

Upon membrane injury, calcium influx triggers the immediate recruitment of annexins to exposed anionic phospholipids around the injury site [184]. This recruitment occurs in a temporally ordered manner, with each annexin exhibiting distinct Ca^2+^ sensitivities and lipid-binding specificities as initially revealed by Annette Draeger and colleagues [185]. For instance, live imaging of laser-induced injuries in zebrafish myofibers revealed that AnxA6 initiates repair by forming a stabilizing patch at the injury site. AnxA2 and AnxA1 are subsequently recruited in a cascade-like process, reflecting the sequential dynamics of the repair machinery [184].

Different annexins contribute distinct functions during repair. AnxA1 and AnxA2, for example, interact with dysferlin, a protein critical for muscle membrane repair, to localize and stabilize it at injury sites [184, 186]. Genetic studies in zebrafish and mouse models identified AnxA6 as a vital player in myofiber repair, with mutations in its gene linked to impaired repair and exacerbated disease [184, 187]. Annexins are consistently recruited to sites of laser-induced injuries, playing critical roles in facilitating muscle repair. Depletion of AnxA1, AnxA2, AnxA5, and AnxA6 impairs the repair process, underscoring their importance in maintaining membrane integrity [188, 189]. Despite their collective involvement, these annexins demonstrate distinct functional specializations. While AnxA2 and AnxA6 are important for the resealing of damaged membranes, AnxA1 primarily contributes to the regeneration of myofibers, highlighting its unique role in the long-term recovery of muscle tissue [190].

**Fig. 3:**
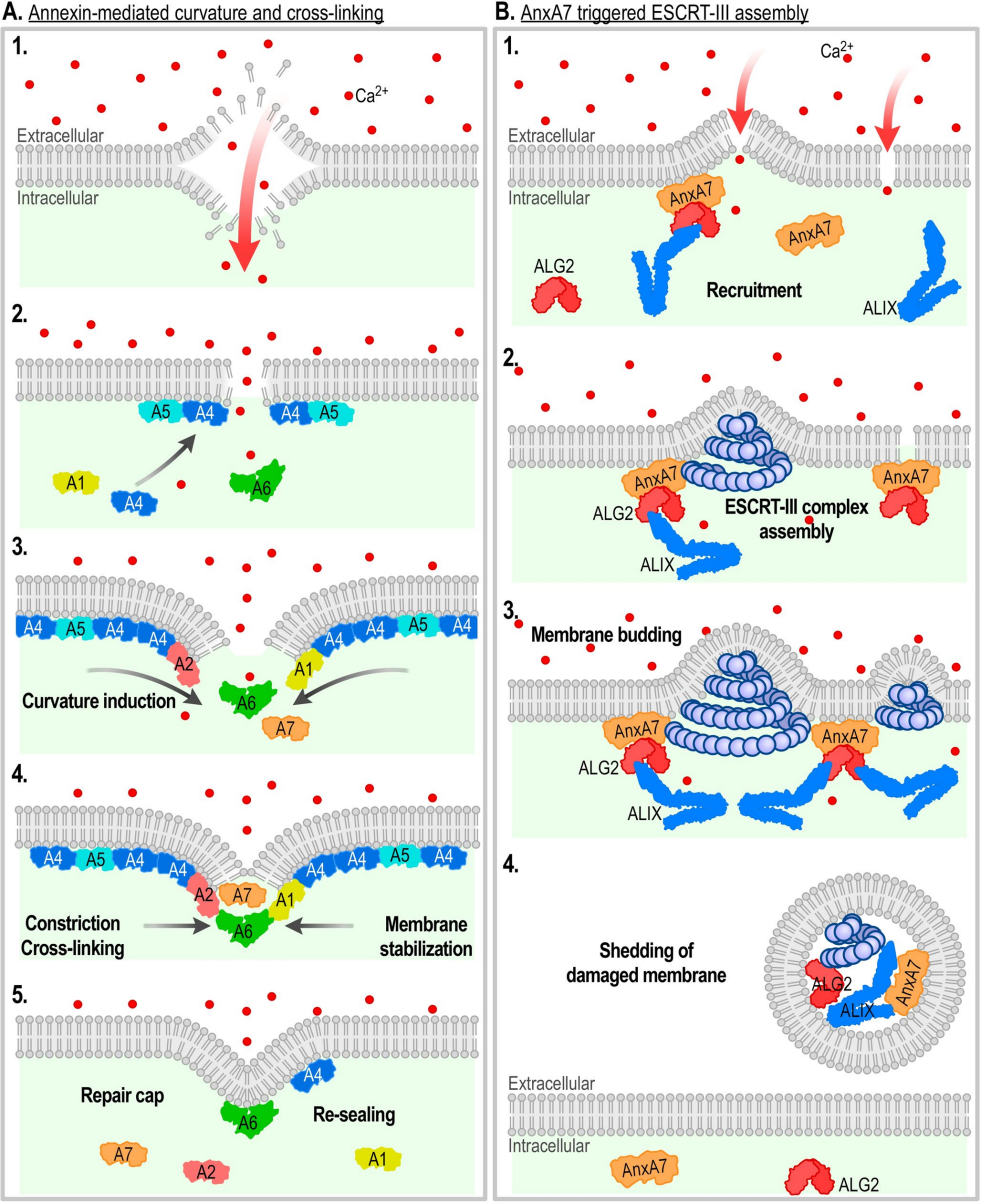
Annexin mediated plasma membrane repair. (**A**) Ca²⁺ influx following plasma membrane injury triggers the recruitment of several annexins to the wound site. Annexins such as AnxA4 and AnxA5 generate forces to curve the free edges of the injured membrane, while AnxA6 produces constriction forces to support membrane resealing. Additional annexins, including AnxA1, AnxA2, AnxA6 and AnxA7, likely support cross-linking and membrane fusion. AnxA4 and AnxA5 form protein lattices that prevent wound expansion. (**B**) Plasma membrane repair involving AnxA7 and Endosomal Sorting Complex Required for Transport III (ESCRT-III). In resting cells, AnxA7, apoptosis-linked gene 2 (ALG-2), and ALG-2 interacting protein X (ALIX) are predominantly cytosolic. Upon membrane injury and Ca²⁺ influx, AnxA7, ALG-2, and ALIX form a complex anchored to the injured membrane via the AnxA7 core domain. ALG-2 and ALIX recruit charged multivesicular body protein 4 (Chmp4) proteins, including Chmp4b, which assemble into spring-shaped ESCRT-III filaments. Contraction of these filaments results in membrane excision and shedding of the damaged membrane. This process likely functions to refine and reorganize the membrane after the initial annexin-mediated repair response. Annexin-mediated curvature and cross-linking, along with AnxA7-induced ESCRT-III assembly and membrane shedding, can accommodate membrane holes of various sizes

Repairing membrane lesions caused by mechanical means or by bacterial pore-forming toxins, also involves the rapid activation of the ESCRT-III machinery. This Ca^2+^-triggered repair pathway facilitates the excision and shedding of damaged membrane fragments to restore integrity [191]. A key player in this process is AnxA7, which binds to ALG-2 upon injury, enabling recruitment of the ESCRT-III adapter protein Alix to the site of damage [191, 192]. This interplay exemplifies the sophisticated Ca^2+^-activated coordination between annexins and ESCRT-III to position the complex correctly at the damaged membrane (Fig. 3).

Mutations in anoctamin 5 (ANO5, or TMEM16E), a member of the TMEM16/ANO family that includes ion channels and phospholipid scramblases, are linked to limb-girdle muscular dystrophy R12, likely due to defective plasma membrane repair. In ANO5 knock-out mice, annexin trafficking at injury sites was altered, interfering with repair cap formation. AnxA2 accumulated to nearly twice the wild-type level, while AnxA1, A5, and A6 showed impaired recruitment. These changes coincided with structural alterations in the annexin repair cap and increased shedding of annexin-positive microvesicles (MVs). The rescue of the repair defect by a scramblase-deficient ANO5 mutant suggests a novel, scramblase-independent function for ANO5, potentially in annexin coordination during repair [193].

Beyond ANO5, annexin-containing MVs play a key role in plasma membrane repair. Following calcium influx triggered by membrane injury, annexins form a scab-like structure at the lesion before being cleaved by calpains, which promotes their secretion within MVs. This process likely helps clear damaged membrane components and facilitates repair [194].

Additionally, exosomes are secreted upon MVB fusion with the plasma membrane, a process also regulated by Ca²⁺-dependent repair. AnxA6 has been identified as a key factor in this mechanism, tethering MVBs to the plasma membrane and promoting exosome release. Cells experiencing frequent membrane stress, such as muscle and metastatic cancer cells, likely increase MV and exosome secretion as part of their repair response, contributing to extracellular vesicle pools in biological fluids [195].

### Annexin-S100 protein interactions in plasma membrane repair

The pivotal role of annexins in membrane repair is significantly amplified by their interactions with S100 proteins. These interactions form complexes that not only stabilize damaged membranes but also coordinate with cytoskeletal remodeling to enhance repair efficiency. For example, the AnxA2-S100A10 complex interacts with the enlargeosome protein AHNAK (desmoyokin), a repair-associated protein, to promote membrane stabilization and facilitate efficient resealing [196]. Similarly, as shown in the Jaiswal and our laboratories, the AnxA2-S100A11 complex is critical for cytoskeletal dynamics in cancer cells, driving F-actin accumulation at injury sites to aid in wound closure and the abscission and clearance of damaged membrane segments [197, 198].

In endothelial cells, S100A11 supports the recruitment of AnxA1 and AnxA2 to injury sites. This Ca^2+^-dependent process involves extended Syt1, which serves as a tether that regulates ER-plasma membrane contact sites [199]. The interaction may form a structural bridge between the injured membrane and the ER, providing the necessary support for membrane restoration [199]. These cooperative interactions emphasize the ability of annexins to integrate membrane stabilization processes with cytoskeletal remodeling, highlighting their multifaceted role in cellular repair mechanisms.

Structural studies have demonstrated that Ca²⁺ binding to S100 proteins, with the exception of S100A10 (which due to mutations in the Ca^2+^ binding loops resides in a permanently active conformation), is a prerequisite for stable S100-annexin interactions. S100 proteins form dimers, thereby presenting in principle two identical annexin binding sites and permitting the formation of heterotetrameric S100-annexin complexes [167]. Such complexes are ideally suited to physically link membrane surfaces bound by the annexin cores assisting membrane closure in the course of plasma membrane wound repair. Of the twenty-five S100 protein family members, seven (S100A1, S100A4, S100A6, S100A10, S100A11, S100A12, and S100B) have been reported to interact with at least one of the twelve human annexin proteins, and some S100 proteins, including S100A6, have been observed to form complexes with various annexin proteins. For instance, AnxA2, A5, A6, and A11 all interact with S100A6 [200]. Thus, the multifaceted role of annexins in Ca^2+^-dependent membrane repair is likely to be regulated by a multitude of interactions with different S100 proteins.

### Lysosomal membrane repair

In addition to the plasma membrane, annexins also contribute to the repair of lysosomal membranes. Lysosomes are membrane-bound organelles filled with hydrolytic enzymes, which play a critical role in cellular degradation and recycling [201]. Repair relies on annexins such as AnxA1, AnxA2 and AnxA7 to restore membrane integrity following injury. Lysosomal membrane damage, resulting in permeabilization and the leakage of Ca²⁺ into the cytoplasm [202], activates annexins, which promptly promote resealing of the membranes and thereby, prevent the release of hydrolytic proteases into the cytoplasm.

AnxA1 and AnxA2 have emerged as important players in lysosome repair, with evidence linking their activity to maintaining lysosomal integrity [203, 204]. In dendritic cells, AnxA2 and its interacting protein S100A10 are recruited to late endosomal and lysosomal membranes when these organelles become destabilized during the phagocytosis of polyethylene particles [203]. This recruitment is thought to aid in membrane stabilization and/or resealing, helping to suppress inflammation by limiting damage-induced immune activation.

Recent studies in human osteosarcoma U2OS cells have further demonstrated that both, AnxA1 and AnxA2 contribute to lysosomal repair [204]. These annexins specifically address larger lysosomal injuries exceeding 4.6 nm, likely working in tandem with the ESCRT machinery. This collaboration is supported by observations that lysosomes labeled with AnxA1 or AnxA2 frequently also show the presence of charged multivesicular body protein 4a, a core ESCRT-III component [204]. However, annexin-mediated repair can also occur independently of the ESCRT-III complex, as observed with AnxA7 [205]. Although AnxA7 is essential for recruiting and positioning the ESCRT-III machinery at the plasma membrane [192], this mechanism is not required at the lysosomal membrane during repair. At damaged lysosomes, AnxA7 is recruited and may facilitate repair by leveraging its abilities to induce membrane curvature and mediate cross-linking [205]. Taken together, these findings highlight the complex and layered nature of lysosome repair mechanisms, which resemble those involved in plasma membrane repair.

Given the location of some of the molecular players of the lysosomal membrane repair kit, the possibility that lysosomal membrane repair could be orchestrated, at least to some extent, from inside the lysosomal lumen should be considered. Membrane repair from the inside may effectively contribute to restore the structural integrity of the lysosomal membrane and might be particularly critical for larger membrane breaches that cannot be quickly sealed by the recruitment of cytosolic annexins alone. Hence, ILV back-fusion appears to be part of a multifaceted lysosomal membrane repair that ensures the continued functionality of the lysosome, especially under stress conditions.

Since annexins can be located in the cytosol or reach the lumen of endolysosomes through mechanisms like CMA or microautophagy (Figure. 2), it is tempting to speculate that these annexins could then bind to the damaged membrane and initiate the repair processes from both sites. For example, AnxA7 was shown to participate in lysosomal membrane repair from the outside [205]. Yet, AnxA7 also harbours KFERQ motifs, which commonly serve as a recognition sequence to target proteins to lysosomes via CMA, thus enabling AnxA7 to be positioned at the surface of ILV to participate in membrane repair from the inside. In addition, annexins may communicate with several other proteins that have been proposed to be involved in this process, possibly even via direct protein-protein interactions. These include lysosome-associated membrane protein 2 A, a key CMA regulator [206]; Syt7, which is involved in Ca^2+^-dependent vesicle fusion and associated with lysosomal exocytosis [207]; and Tsg101, which is a key component of the ESCRT machinery required for membrane repair, and found associated with Alix, CD63, and represents a major protein marker of exosomes [75].

Finally, other proteins at the cytosolic side of lysosomes might also take part in the repair process. For instance, the cytoskeletal protein cortactin that assists actin polymerization also localizes to damaged lysosomal membranes and may work in concert with annexins to facilitate membrane stabilization and fusion processes [208]. In fact, Rab GTPases, in particular the late endosomal/lysosomal Rab7, coordinate the vesicular transport and membrane fusion that could be critical for membrane repair, especially when considering back-fusion of intraluminal vesicles [209]. In this context, the scaffolding function of AnxA6 to recruit the Rab7-GAP TBC1D15 (see Chap. 2.1.2) to LE/Lys could be relevant, the latter having a prominent role in acute lysosomal membrane damage repair and lysosomal regeneration [210].

Hence, one cannot entirely rule out the existence of a fourth population of ILVs harbouring molecules committed to lysosomal membrane repair “from the inside” (e.g. annexins) (Fig. 2). The hypothesis that multiple ILV populations can coexist within a single MVB is substantiated by the notion that the ultimate destination of a particular MVB is subject to variation, depending on the prevailing physiological requirements at a specific timepoint of the endolysosomal lifespan and related to degradation, exosome production, housekeeping or membrane repair.

### Mechanisms of annexin-mediated remodeling

Annexins perform their repair functions by cross-linking membranes, structural stabilization and by inducing membrane curvature. Members of the annexin family possess a distinct ability to induce membrane curvature, a characteristic initially observed by our laboratories in double-supported membrane models with exposed free edges [182]. Specific annexins, such as AnxA4, A5 and A6, can generate different curvature morphologies through Ca^2+^-mediated binding to anionic lipids in the membrane. During wound healing, AnxA4 monomers are thought to organize into trimers, inducing out-of-plane curvature to facilitate membrane closure. Similarly, AnxA6, with its distinctive two-core structure, is believed to constrict around larger lesions, compressing and stabilizing the damaged membrane [182, 183].

To further support repair, AnxA5 forms two-dimensional arrays at wound sites, creating a scaffold that limits wound expansion and facilitates protein recruitment as shown by the Brisson/Bouter laboratory [211, 212]. Annexins also exhibit feedback mechanisms, as some annexins have an affinity for highly curved membrane regions [178]. AnxA4 is found to be a sensor of negative membrane curvatures, which upon injury and annexin-mediated bending recruits additional annexins such as AnxA4 to amplify the repair response [178]. These processes illustrate the dual role of annexins as both structural stabilizers and dynamic remodelers.

Several unresolved questions remain and future studies will need to clarify how annexins may discriminate between injury types. Also, despite many live cell imaging in this field, it remains to be determined if solely Ca^2+^ signals, or if lipid compositions and membrane properties contribute to guide recruitment of annexins to injured membrane sites. Future research should also explore whether annexins can form molecular structures to temporarily seal membrane breaches. Addressing these gaps will advance our understanding of annexin biology and facilitate the development of targeted therapies.

## Annexins and their RNA-binding properties in membraneless organelles

Although annexins are traditionally known for their role in membrane dynamics, certain annexins have also been shown to bind RNA, expanding their functional repertoire [213]. This property is especially notable for annexins AnxA2 and A11, which have been implicated in affecting RNA transport, stability and translation [214, 215]. In addition, annexins directly or indirectly associated with RNAs can be transferred between cells in exosomes. Together with the ability of annexins to recruit RNAs including miRNAs into exosomes, this suggests that they could function in the control of cell-cell interactions, thereby affecting the adaptive responses and remodelling processes during disease [216] (also see Chap. 2.1.2. and Fig. 2).

Some membraneless organelles are defined as subcellular compartments that lack a surrounding membrane. These structures are characterized by the dynamic assembly of proteins and nucleic acids, and are formed through a process known as liquid-liquid phase separation, resulting in the creation of condensates. Examples for these specialized microenvironments with unique biochemical functions include the nucleolus, P bodies, nuclear and paraspeckles, stress and RNA granules.

Interestingly, several annexins appear to have the capacity to bind membrane-less organelles. A pioneering study showed that AnxA11 acts as an anchor mediating the association of RNA granules with lysosomes during their transport to the distal regions of the axon [170]. While the C-terminal domain of AnxA11 with the conserved annexin repeats confers the ability to bind lysosomes, the long N-terminal AnxA11 domain allows intercalation within phase-separated RNA granules. Several AnxA11 mutations have been directly linked to amyotrophic lateral sclerosis, either interfering with the ability of AnxA11 to interact with RNA granules (N-terminal mutations) or lysosomes (C-terminal mutations), respectively. Consequently, both types of mutations compromise the coupling between RNA granules and lysosomes, disrupting RNA granule transport. Hence, the tethering function of AnxA11 represents a new mechanistic and structural link between lysosomes and a membraneless compartment in ALS pathogenesis (see also Chap. 4). The capacity of AnxA11 to ensemble with ribonucleoproteins on lysosomes has been dissected in a recent study. Here, ALG2 and calcyclin (S100A6) were identified as crucial regulators of AnxA11-based phase coupling, adding to a better understanding of the juxtaposition of biomolecular condensates and organellar membranes [217].

Alike the N-terminus of AnxA11 [170], the AnxA7 N-terminal domain is rich in proline residues and may behave like other well-characterized domains that undergo phase separation and/or fibrillization [213]. In addition, the ability of annexins to bind mRNA, as identified in Anni Vedeler’s laboratory initially for AnxA2 [218], might contribute to annexins interacting with RNA-containing membraneless organelles. These findings might reflect a common theme amongst annexins, as all rat annexins can bind RNA and messenger ribonucleoprotein complexes [213]. At least in the case of AnxA2, this supports transport and localized translation of specific mRNAs, such as c-myc, which critically controls cell proliferation [213, 214].

Other annexins may also be capable of associating with membraneless RNA-containing organelles such as AnxA10, which was identified as part of paraspeckles [219, 220]. Furthermore, the formation of homo- and heterogeneous annexin assemblies observed previously may indicate that interactions between multiple annexins, as previously proposed [221, 222], may contribute to the tethering of (pre-)lysosomal compartments to other organelles.

As mentioned above, AnxA6, possibly in conjunction with AnxA2 and S100B (see below), interacts with the N-terminus of the tau protein. This region of the tau protein is responsible for the generation of tau liquid-liquid phase separated structures that have been described to be responsible for tau’s axonal localization [155, 223, 224]. However, the aggregation of tau into amyloid filaments and the liquid-to-solid transition of tau droplets/condensates [225] inside neurons is the main hallmark of tauopathies (i.e. Alzheimer’s disease). We speculate that besides AnxA6, several other interactors of tau that regulate the liquid-liquid phase separation also bind to AnxA6, for example S100B [226, 227], with roles in tau-related pathologies [155, 228].

## Conclusions

As outlined in this review, a vast body of evidence has linked annexins to distinct steps of endo- and exocytotic membrane traffic, the organization of membranes and their repair, as well as the establishment and regulation of specialized membrane domains. These well-recognized functions nicely concur with the fundamental property shared by almost all annexins, their Ca^2+^-sensitive binding to acidic phospholipids of cellular membranes. Thereby, members of this family are suited ideally to fulfill regulatory functions in the membrane steps in question, in particular those that are dependent on local or global Ca^2+^ mobilization. Importantly, with the evolution of the annexin-type of membrane binding, cells have established a membrane binding module that can react immediately to rapid Ca^2+^ changes by membrane association resulting in the formation of platforms that can recruit other factors and prepare membranes for efficient fusion and fission events. This annexin function is particularly evident in membrane repair where many members of the family come together as part of a fundamentally important machinery executing membrane resealing and thereby permitting cell survival. The central role of annexins in membrane repair also makes them promising therapeutic targets for disorders involving defective repair, such as muscular dystrophies and metastatic cancers. Enhancing annexin activity may alleviate muscle degeneration, while targeting annexin-mediated repair in cancer cells could sensitize them to therapeutic agents.

## Data Availability

NA.
